# Large B-cell lymphoma mimicking iliopsoas abscess following open revision of proximal femur infected non-union: a case report

**DOI:** 10.1186/1756-0500-7-470

**Published:** 2014-07-23

**Authors:** James McCammon, Randy Mascarenhas, Michael J Monument, Abdul Elyousfi, Brad Pilkey

**Affiliations:** 1 Section of Orthopedic Surgery, Health Sciences Center, University of Manitoba, Winnipeg, Manitoba, Canada; 2 Rush University Medical Center, Chicago, IL, USA; 3 Department of Orthopedics, University of Utah, Salt Lake City, UT, USA; 4 Department of Orthopedic Surgery, University of Manitoba, 720 McDermot Ave, AD420 Health Science Centre, Winnipeg, MB R3T 2N2, Canada

**Keywords:** Lymphoma, Infection, Antigen stimulation, Metal implants

## Abstract

**Background:**

Extranodal presentation of lymphoma is a rare occurrence. It has been postulated that chronic antigen stimulation may predispose a patient to the development of lymphoma.

**Case presentation:**

We present a case report of a large extranodal B-cell lymphoma mimicking a postoperative abscess following surgery for an infected proximal femur nonunion in an 80-year-old Caucasian male of Italian descent.

**Conclusions:**

This case highlights the need to consider malignancy in revision surgery, careful examination of operative specimens and the need for further understanding of the role of metal implants in chronic antigen stimulation.

## Background

Lymphomas can develop in any location where lymphomatous tissue exists, but extranodal presentation in the lower limb and pelvis are uncommon. We report an unusual case of large extranodal B-cell lymphoma initially presumed to be a postoperative pelvic and thigh abscess following multiple operations for an infected proximal femur nonunion.

## Case presentation

An 80-year-old Caucasian male of Italian descent presented to the emergency room with a two-week history of progressive left thigh pain and swelling. The patient denied any history of trauma and stated that the pain was aggravated by weight-bearing. He denied any fevers, chills, night sweats, constitutional or neurological symptoms. He had no drug allergies and his only medication was a statin. Past medical history included hypercholesterolemia, open cholecystectomy, and prostate cancer requiring resection remotely in the past.

**Figure 1:**
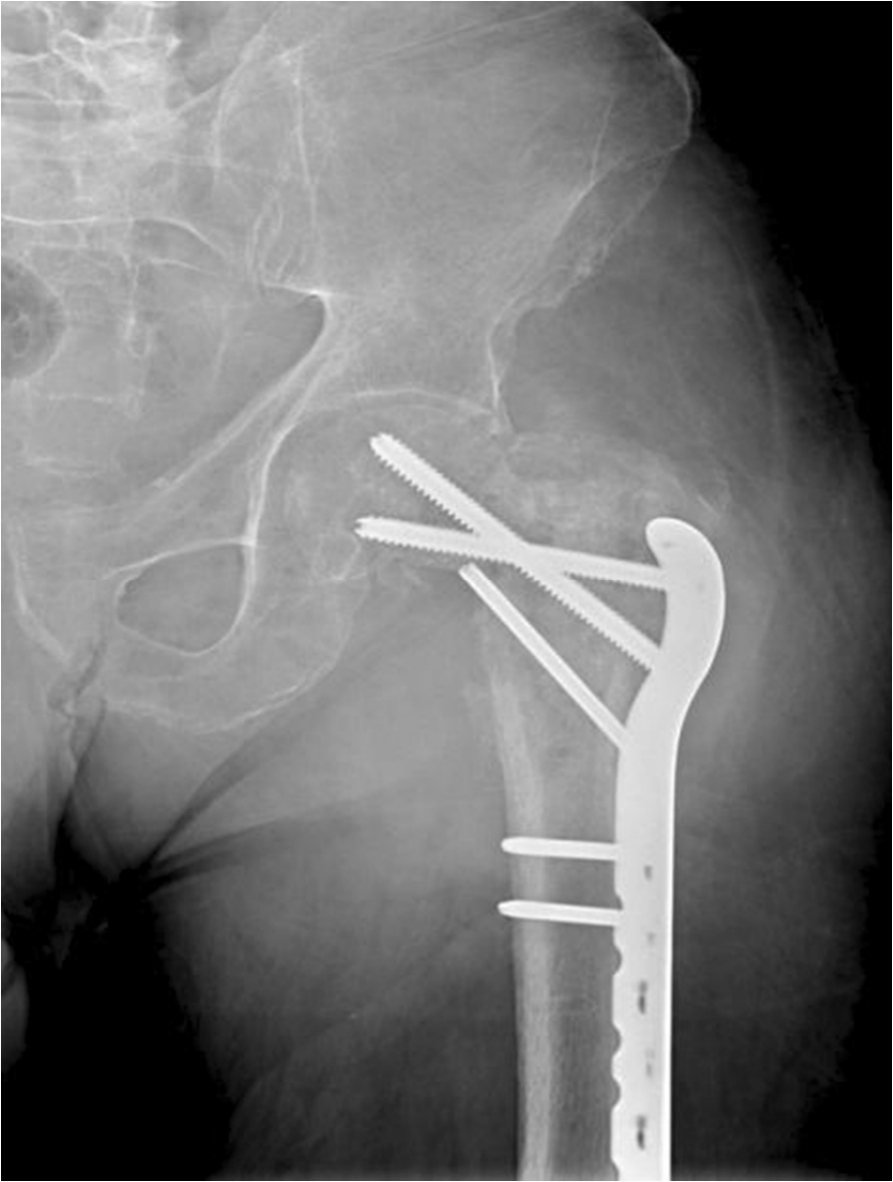
**Anterior-posterior radiograph of the left hip.** This shows a proximal femoral locking plate in good position with moth-eaten appearance of proximal femur and surrounding lysis that could be suggestive of infection or malignancy.

**Figure 2:**
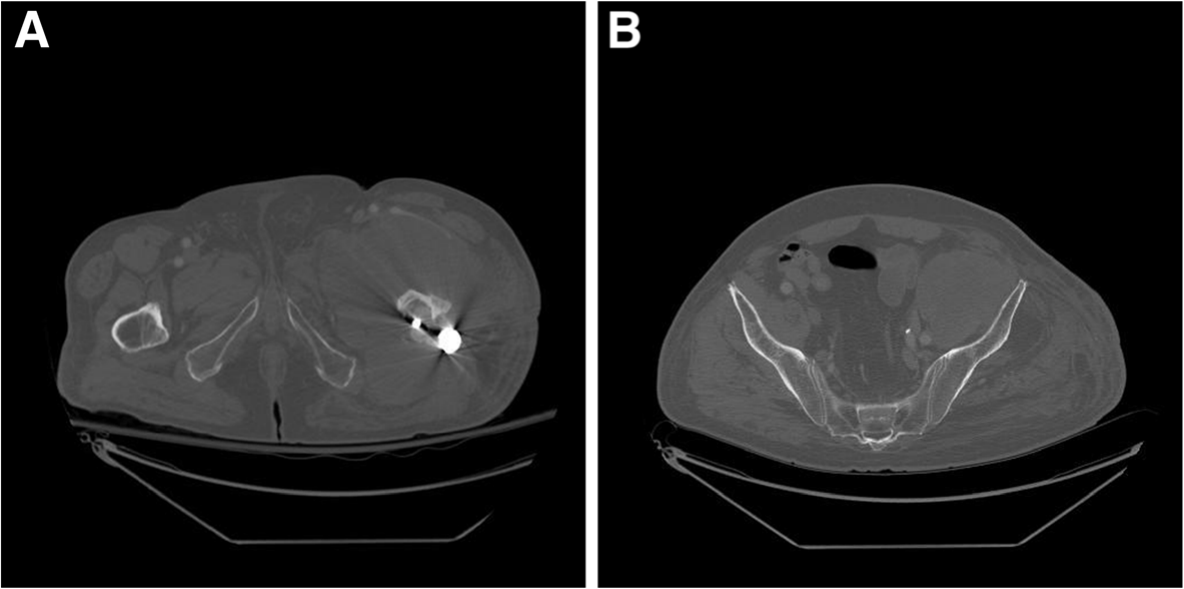
**Axial computed tomography of pelvis. A**: A dense collection/mass in anterior left thigh with lateral extension at level of the proximal femur. **B**: Additional cuts revealing that the collection/mass extends to the pelvis.

**Figure 3:**
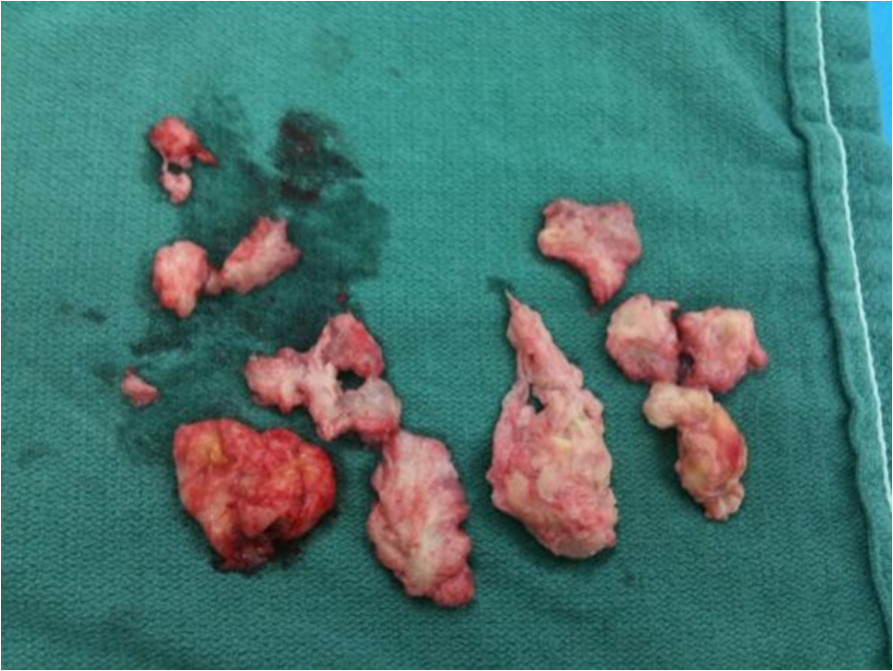
**Intra-operative specimen.** These samples were taken at the time of initial incision and drainage. Cultures were negative while pathology came back positive for B-cell lymphoma.

The patient’s surgical history was significant for six previous operations for a left intertrochanteric femoral fracture complicated by an infected non-union. An infectious diagnosis had been proven with *Staphylococcus aureus* cultured from previous operations. His most recent surgery occurred one month prior to his presentation to the emergency room and involved removal of a left trochanteric fixation nail and excision of a pseudoarthrosis, followed by an osteotomy of the left proximal femur nonunion and fixation with a proximal femoral locking plate (Figure 1). No complications were encountered during that surgery or afterwards, and the patient had been discharged home several days after surgery with instructions to avoid bearing weight on the left leg.On physical examination, the patient was hemodynamically stable and afebrile. A general physical examination, including lymph nodes, was unremarkable. Inspection revealed pronounced firm swelling of the left thigh with slight erythema. The surgical incision on the lateral aspect of the left thigh was clean and dry. There was slight tenderness to palpation over the swollen area and left hip and knee range of motion was restricted secondary to pain in the left thigh. The patient was neurovascularly intact in his left leg. Lab results, including erythrocyte sedimentation rate, C-reactive protein, white blood cell count, and hemoglobin levels were all within normal range. Left hip radiographs showed only generalized soft tissue swelling of the left thigh with no signs of hardware failure. Computed tomography (CT) scanning of the pelvis and left thigh was suggestive of the diagnosis of a psoas abscess (Figure 2A, B).After obtaining blood and urine cultures, the patient was admitted to hospital and broad-spectrum intravenous antibiotics were started based on recommendations from a surgical infectious disease specialist. The patient underwent incision and drainage for the left thigh abscess that same day using the previous lateral hip incision. No pus was noted intraoperatively, but a collection of thick white gelatinous tissue was noted underneath the muscle fascia of the left thigh anterior compartment and was observed to extend medially and laterally (Figure 3). Tissue specimens were sent for gram stain, aerobic and anaerobic cultures, acid fast testing, fungal cultures and cytology. The wound was then irrigated with normal saline and antibiotic beads and a hemovac drain were placed inside the wound. Intravenous antibiotics were resumed post-operatively and all cultures were negative. When swelling did not resolve within a few days after surgery, a repeat CT of the pelvis showed residual features of the presumed iliopsoas abscess extending into the left thigh. On postoperative day two, the patient underwent a second look incision and drainage through a combined ilioinguinal and lateral hip approach. A mass was noted close to the left iliac wing encasing the left femoral nerve. The femoral nerve was freed from the lesion without complication and the mass was then removed, debulked, and sent for frozen section. The frozen section revealed acute and chronic inflammation. The remaining tissue was sent for microbiology and histopathology. The wound was irrigated with normal saline and a hemovac drain was again placed before wound closure. Postoperatively, the patient was re-evaluated by the surgical infectious disease service, who proceeded to discontinue IV antibiotics and advised initiating anti-tuberculosis therapy. This decision was based on a diagnosis of exclusion, given the negative bacterial cultures, the anatomic site of the abscess and the gross pathological features of the biopsied tissue. All screening tests for tuberculosis were negative. CT scans of the chest and abdomen were performed as part of a metastatic work up, but revealed only a large right-sided kidney cyst. The patient improved and was discharged home with oral anti-tuberculosis therapy to be taken until definitive pathology results were available. Two weeks later, final pathology reports supported the diagnosis of a diffuse large B-cell lymphoma. Anti-tuberculosis drugs were discontinued and a medical oncologist was consulted to initiate chemotherapy.

### Discussion

Diffuse large B-cell lymphoma is the most commonly occurring lymphoma, estimated to account for 35% of all lymphoma cases globally [1]. It typically presents in patients older than 60 years, has an aggressive natural history, and is primarily treated with systemic chemotherapy [2]. They typically develop in nodal tissue with less common development in extra-nodal tissue with estimates of approximately 1.6% [3]. Extra-nodal sites include the gastrointestinal tract, orbit, respiratory tract, skin, bone, thyroid, central nervous system and (less commonly) the soft tissues [4,5].

Lymphomas can also involve any part of the musculoskeletal system and occasionally can manifest as a primary soft tissue mass that can be mistaken for infection or a soft tissue sarcoma [6-8]. Soft tissue lymphoma accounts for approximately 1% of malignant soft tissue neoplasms and are much more likely to present as non-Hodgkin’s lymphoma rather than Hodgkin’s lymphoma [3]. Most patients presenting with soft tissue lymphomas are older than 50 years, with no predilection to either sex [6].

Although poorly understood, it has been postulated that primary soft tissue lymphomas may be the result of chronic antigen stimulation in an individual with a deficient immune system. In addition to inflammatory connective tissue disorders and viruses, other potential causes of a prolonged local inflammatory environment in patients undergoing orthopedic surgery include shedding of local metal ions and debris from metallic implants, radiation from radiographs and CT imaging, surgical trauma and infection.

Pre-clinical evidence examining the potential carcinogenic properties of metal implants found a trend towards sarcoma development in rats after 30 months when implants with a high content of cobalt, chromium or nickel were used. There was also an increase in bone-associated lymphoma development in rats with these metal implants [9]. It is believed that the local accumulation of metallic debris can cause damage to the cell membrane of macrophages with subsequent release of pro-inflammatory mediators as well as the formation of immunogenic complexes, both of which can stimulate immune system responses [10,11].

Numerous studies have explored the topic of increased cancer risk in individuals with metal implants, but have shown discordant conclusions. Gillespie et al. [12] followed a cohort of total hip arthroplasty (THA) patients and found that the incidence of lymphatic and hematopoietic malignancies was nearly twice that expected for the general population. Similarly Visuri and Koskenvuo [13] also looked at THA patients and observed an incidence of lymphomas and leukemias that was three times what was expected. However, more recent studies have shown no increase in risk of cancer [14-16]. There are approximately thirteen recorded instances of lymphoma at sites associated with metal implants in the literature (Table 1).

**Table 1:** Case reports of lymphoma in patients with metal implants

**Author, year**	**Age (years)/Gender**	**Implant**	**Time to presentation (years)**	**Symptoms**	**Diagnosis**	**Treatment**
McDonald [17], 1981	48/M	Cobalt chromium plate and screws in tibia	17 years	Aching, night sweats, malaise	Histiocytic lymphoma	Chemotherapy and Radiotherapy
Dodion et al. [18], 1983	50/M	Cobalt chromium screw and plate	14 months	Local pain	Diffuse Large B-cell lymphoma	Chemotherapy and Radiotherapy
Syed et al. [19], 1997	75/F	Exeter Hip Replacement	7 years	Increasing thigh and groin pain with inability to weight bear	Non-Hodgkins Lymphoma	Radiotherapy
Rahdi et al. [10], 1998	25/M	Internal fixation of distal tibia	8 years	Slow growing ankle mass	Diffuse Large B-cell lymphoma	Chemotherapy, Below-knee amputation
Rahdi et al. [10], 1998	64/F	Primary Total Hip	4 years	6 month history of pain and swelling in thigh	Diffuse Large B-cell lymphoma	Chemotherapy and Radiotherapy
Ito et al. [20], 1999	80/F	Primary Total Hip	8 years	Pain in hip	Diffuse Large B-cell lymphoma	Radiotherapy
Ganapathi et al. [21], 2001	85/M	Primary Total Hip, Revision Total Hip	12 years post THA, 10 years post revision, 14 mo post periprosthetic fracture	Chronic draining sinus, anorexia, lethargy, drowsiness	Diffuse Large B-cell lymphoma	Died prior to radiotherapy
O’Shea et al. [22], 2006	75/F	Primary Total Hip	13 years	Chronic draining sinus, pain and swelling of thigh	Diffuse Large B-cell lymphoma	Chemotherapy, radiotherapy, excision arthroplasty
Hsieh et al. [23], 2007	30/F	Primary Total Hip	4 years post	3 month history of hip pain	Diffuse Large B-cell lymphoma	Chemotherapy
Cheuk et al. [24], 2008	78/M	Primary Total Hip, Revision × 2	32 years post primary TKR, 16 years post revision	Increasing knee pain	Diffuse Large B-cell lymphoma	Radiotherapy
Eskander et al. [25], 2008	70/F	Primary Total Knee	1 year	Two superficial areas of skin necrosis as well as surrounding ecchymosis and edema	Diffuse Large B-cell lymphoma	Radiotherapy, knee fusion
Palraj et al. [26], 2010	77/M	Stainless screw plate and screws for tibial fracture	7 years	2 weeks of pain and erythema, localized swelling, minimal warmth	Anaplastic T-cell lymphoma	Chemotherapy
Chaudhry et al. [27], 2011	76/M	Primary Total Knee	3 years	1 week history of knee pain	Diffuse Large B-cell lymphoma	Chemotherapy and Radiotherapy

*Abbreviations*: *M* male, *F* female, *TKR* total knee replacement, *THA* total hip arthroplasty.

Chronic inflammation as a result of infection is a possible contributing factor in the development of lymphoma, as implied by studies of non-infectious consequences of osteomyelitis [28-31]. Cheuk et al. [24] also make this link, arguing that lymphoma associated with osteomyelitis shares characteristics of that associated with metallic implants. These characteristics include the fact that these solid tumors arise in the context of chronic inflammation, occur in a confined space, and have a long latency period between the onset of inflammation and the development of lymphoma.

Typically, soft tissue lymphomas present in patients older than 60 years as enlarging soft tissue masses associated with swelling or pain. In typical cases, there are usually no B symptoms such as weight loss and fever [4,6]. Our case features a similar presentation with an enlarging thigh mass associated with pain with the absence of B symptoms. Although the patient showed no identifiable active cause of immunocompromise, he had several risk factors for chronic antigenic stimulation including several surgeries involving metallic implants and prolonged infection after his index surgical procedure.

Despite the presence of some risk factors, the malignancy in this case could also have been coincidental. Cases have been reported of patients who underwent total joint replacement and were found to have lymphoma on routine histopathological exams [32,33]. One study looking at retrieved femoral heads that were to be used for bone allograft found that 14 of 852 heads were highly suspicious for low-grade B-cell lymphoma. With a median of 7.2 years follow-up, one patient developed a B-cell lymphoma in a lymph vessel in the inguinal region on the contralateral side [34].

## Conclusion

This case underlines the importance of considering the possibility of malignancy with revision surgery, particularly in cases that present as infectious in nature. It also re-affirms the importance of routine pathologic examination of operative specimens. As the number of joint replacements and fixation of fractures increase with life expectancy, potential long-term adverse effects of metal implants need to be further investigated.

## Consent

Written informed consent was obtained from the patient for publication of this Case Report and any accompanying images. A copy of the written consent is available for review by the Editor-in-Chief of this journal.

## Competing interests

The authors declare that they have no competing interests.

## Authors’ contributions

BP was the surgeon involved in the operations and follow-up of this patient and conceived of the report; AE contributed to data acquisition; MM contributed the data on lymphoma and was involved in editing the drafts; RM and JM contributed to data acquisition, drafting and editing the manuscript. All authors read and approved the final manuscript.
